# Identification of differentially expressed genes in diabetic kidney disease by RNA‐Seq analysis of venous blood platelets

**DOI:** 10.1002/2211-5463.13199

**Published:** 2021-07-02

**Authors:** Bao Long Zhang, Xiu Hong Yang, Hui Min Jin, Xiao Li Zhan

**Affiliations:** ^1^ The Institutes of Biomedical Sciences (IBS) Fudan University Shanghai China; ^2^ Division of Nephrology Pudong Medical Center Shanghai Pudong Hospital Fudan University Shanghai China

**Keywords:** chronic kidney disease, diabetic kidney disease, GDTLs, KCND3, platelet RNA‐Seq

## Abstract

Diabetic kidney disease (DKD) is the leading cause of end‐stage renal disease. However, because of shared complications between DKD and chronic kidney disease (CKD), the description and characterization of DKD remain ambiguous in the clinic, hindering the diagnosis and treatment of early‐stage DKD patients. Although estimated glomerular filtration rate and albuminuria are well‐established biomarkers of DKD, early‐stage DKD is rarely accompanied by a high estimated glomerular filtration rate, and thus there is a need for new sensitive biomarkers. Transcriptome profiling of kidney tissue has been reported previously, although RNA sequencing (RNA‐Seq) analysis of the venous blood platelets in DKD patients has not yet been described. In the present study, we performed RNA‐Seq analysis of venous blood platelets from three patients with CKD, five patients with DKD and 10 healthy controls, and compared the results with a CKD‐related microarray dataset. In total, 2097 genes with differential transcript levels were identified in platelets of DKD patients and healthy controls, and 462 genes with differential transcript levels were identified in platelets of DKD patients and CKD patients. Through Kyoto Encyclopedia of Genes and Genomes pathway enrichment analysis, we selected 11 pathways, from which nine potential biomarkers (*IL‐1B*, *CD‐38*, *CSF1R*, *PPARG*, *NR1H3*, *DDO*, *HDC*, *DPYS* and *CAD*) were identified. Furthermore, by comparing the RNA‐Seq results with the GSE30566 dataset, we found that the biomarker *KCND3* was the only up‐regulated gene in DKD patients. These biomarkers may have potential application for the therapy and diagnosis of DKD, as well aid in determining the mechanisms underlying DKD.

## Graphical abstract

Based on platelet RNA sequencing of venous blood platelets, we identified 2097 genes with differential transcript levels in platelets of patients with diabetic kidney disease compared to patients with chronic kidney disease and 462 genes with differential transcript levels compared to healthy controls, and found nine potential biomarkers, with KCND3 identified as the most promising biomarker for clinical diagnosis of diabetic kidney disease.

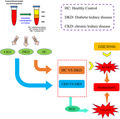

**Abbreviations**
AUC: area under the curve
BP: biological processes
CC: cellular components
CKD: chronic kidney disease
DEG: differentially expressed gene
DKD: diabetic kidney disease
DM: diabetes mellitus
eGFR: estimated glomerular filtration rate
ESRD: end‐stage renal disease
GBM: glomerular basement membrane
GDTLs: genes with differential transcript levels
GO: Gene Ontology
GPI: glycosylphosphatidylinositol
HC: healthy controls
JAK-STAT: Janus kinase-signal transducer and activator of transcription
KEGG: Kyoto Encyclopedia of Genes and Genomes
LXR α: liver X receptor α
MF: molecular function
mTOR: mammalian target of rapamycin
NDKD: non-diabetic kidney disease
PPAR: peroxisome proliferator-activated receptor
PPI: protein–protein interaction
RNA-Seq: RNA sequencing
ROC: receiver operating characteristic
TNFR1/2: tumor necrosis factor receptors ½

Chronic kidney disease (CKD) is highly prevalent, with 850 million individuals suffering from the disease and one in 10 adults possibly being at risk worldwide [1]. In addition, CKD patients with renal insufficiency are associated with a higher morbidity and mortality of cardiovascular disease [2]. The main causes of CKD are varied, including diabetes, chronic glomerulonephritis, chronic pyelonephritis and hypertension, amongst others [3]. Up to 75% of CKD patients have increased cardiovascular disease and infection before renal replacement therapy [4]. Therefore, patients with decreased renal function must be monitored and treated effectively to prevent the progression of CKD into end‐stage renal disease (ESRD) [5]. Diabetic kidney disease (DKD), or diabetic nephropathy, occurs in approximately 40% of diabetic patients and is the leading cause of ESRD, accounting for the high morbidity/mortality of cardiovascular diseases worldwide [6]. Between 1990 and 2012, the number of deaths attributable to DKD increased by 94% [5]. Poor blood glucose control often leads to the occurrence and development of DKD, in conjunction with the change of glomerular feedback, the abnormality of polyol metabolism and the formation of advanced glycation end products.

Diabetic kidney disease occurs in patients with diabetes mellitus (DM). Different from primary CKD or non‐diabetic kidney disease in patients with DM, the clinical diagnosis of DKD is based on the presence of proteinuria and impaired kidney function in the setting of diabetes, with a distinct histopathological pattern of glomerular basement membrane (GBM) thickening, mesangial matrix expansion, nodular glomerulosclerosis and arteriolar hyalinosis [7, 8, 9]. Clinical biomarkers such as glomerular filtration rate (eGFR) and albuminuria, which are commonly used for monitoring CKD progression, are not accurate in the diagnosis of DKD or as a reflection of disease progression in DKD patients, especially in the very early stage. Therefore, highly sensitive biomarkers are urgently needed for DKD diagnosis and therapies.

Advances in techniques make it possible for researchers to identify new biomarkers in DKD. Accordingly, a transcriptome analysis of kidney samples of DKD patients was performed by Woroniecka *et al*.[10] aiming to identify differentially expressed genes (DEGs) compared to healthy controls (HCs) via a microarray. Subsequently, several companion studies showed that the inflammation pathway is associated with DKD, with the tumor necrosis factor receptors 1/2 (TNFR1/2) and glycated hemoglobin A1c being remarkable biomarkers for the progression of advanced DKD [11, 12, 13]. In recent years, next‐generation sequencing technology, including high‐throughput RNA sequencing (RNA‐Seq), has developed rapidly [14, 15]. Compared to microarray technology, RNA‐Seq can be used to quantify single‐nucleotide resolution transcripts in the whole genome, thus detecting incomplete species in the genome [16, 17]. RNA‐Seq also has the characteristics of a high signal‐to‐noise ratio and wide application, which makes it an important experimental method for analyzing DEGs at the transcriptional level [18].

However, there are few studies employing RNA‐Seq of platelets in the blood of patients with CKD and DKD. A study has shown that there is overactivation of platelets in patients with diabetes, and the subsequent released platelet products can cause damage to the blood vessel wall and microcirculatory bed (including the kidney) [19]. Furthermore, microalbuminuria observed in preclinical DKD may also be related to platelet activation [20]. Therefore, platelet RNA‐Seq may be a good way of investigating the potential mechanism of DKD and providing better therapies for DKD patients.

In the present study, RNA‐Seq was performed to characterize the transcriptome profiles in the platelets of healthy individuals and patients with CKD and DKD. In total, 2097 genes with differential transcript levels (GDTLs) were identified in HCs compared to DKD patients, and 462 GDTLs were identified in CKD patients compared to DKD patients. To evaluate the changes in gene splicing and signaling pathways, we carried out Gene Ontology (GO) terminology to predict the potential function of these DEGs, and Kyoto Encyclopedia of Genes and Genomes (KEGG) analysis to evaluate the potential regulatory pathways of these DEGs. We identified 11 pathways and nine potential biomarkers: *IL‐1B*, *CD‐38*, *CSF1R*, *PPARG*, *NR1H3*, *DDO*, *HDC*, *DPYS* and *CAD*. Furthermore, we compared the RNA‐Seq data with the microarray data set related to CKD. *KCND3* is the only up‐regulated genes in DKD patients. In sum, we have identified many potential molecular targets and signal pathways, providing a potential and comprehensive theoretical basis for the treatment and diagnosis of DKD patients.

## Materials and methods

### Ethical approval and informed consent

**Fig. 1:**
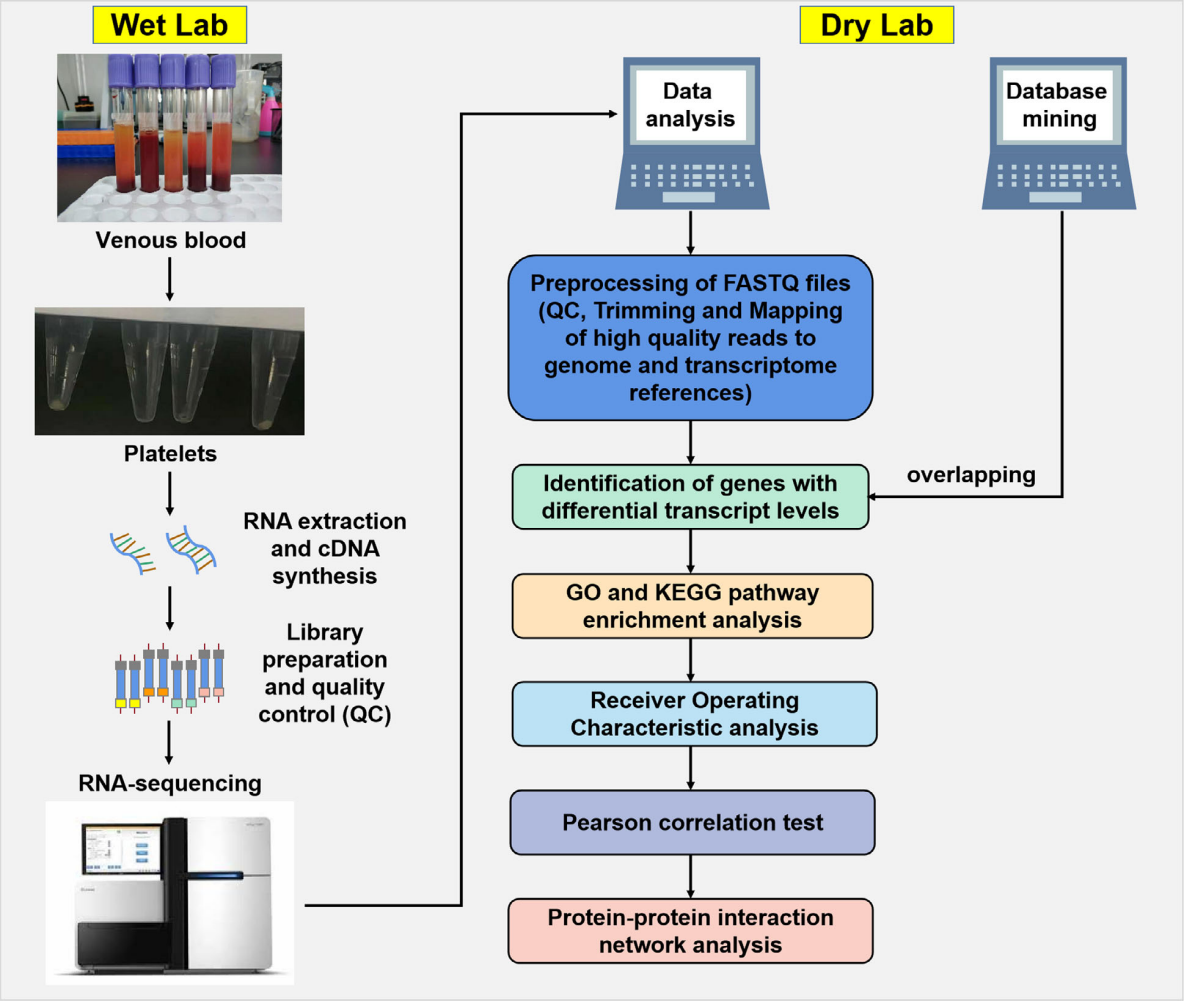
Platelet RNA‐Seq workflow and the bioinformatics analysis steps.

The overall research route is oulined in Fig. 1. None of the patients who participated in this study had a genetic history or other complications. Ten healthy individuals, three CKD patients (the primary disease was chronic glomerulonephritis) and five DKD patients were recruited at Shanghai Pudong Hospital between May 2017 and February 2018. DKD was defined clinically by the presence on two occasions of a ratio of urinary albumin to urinary creatinine from a first morning specimen of at least 300, or albuminuria >300 mg/24 h, or by a 24 h urinary protein concentration > 500 mg, accompanied by diabetic retinopathy. Patients were excluded if they had received a diagnosis of type 1 diabetes or non‐diabetic renal disease. This study was approved by the ethics committee of Shanghai Pudong Hospital and written informed consent was obtained from all subjects. The study was conducted according to the guidelines set by the Declaration of Helsinki. The characteristics of the 18 subjects are summarized in Tables 1 and 2.

**Table 1:** Clinical characteristics of CKD and DKD patients, as well as HCs.

Characteristics	DKD (*n* = 5)	CKD (*n* = 3)	HC (*n* = 10)
Gender (male/female)	2/3	3/0	7/3
Age (mean ± SD)	68.60 ± 7.20	73.00 ± 2.16	58.90 ± 9.09
Diabetic during (year)	8 ± 3	–	No
Kidney function (stage)	Stage 4	Stage 4	No
Glucose‐lowing medication	Insulin	–	No
Antihypertensive drug	ARB	ARB	No
Lipid‐lowing therapy	No	No	No
Aspirin prescribed	No	No	No

**Table 2:** Clinical information.

Disease staging	Sample name	Gender	Age	Group
NA	HC01	Female	73	HC
NA	HC02	Male	73	HC
NA	HC03	Male	64	HC
NA	HC04	Female	52	HC
NA	HC05	Male	54	HC
NA	HC06	Male	50	HC
NA	HC07	Male	60	HC
NA	HC08	Male	52	HC
NA	HC09	Male	65	HC
NA	HC10	Female	46	HC
Stage 4	CKD01	Male	70	CKD
Stage 3	CKD02	Male	74	CKD
Stage 4	CKD03	Male	75	CKD
IV1	DKD01	Female	77	DKD
V3	DKD02	Male	60	DKD
V2	DKD03	Female	74	DKD
II1	DKD04	Male	72	DKD
V1	DKD05	Female	60	DKD

### Platelet isolation

Blood samples drawn from peripheral veins were stored in EDTA‐containing tubes. Platelet‐rich plasma was first obtained via centrifugation at 114 ***g*** for 10 min at 4 °C and then centrifuged again to further remove hemocytes. The supernatant was transferred to a 1.5‐mL Eppendorf tube and centrifuged at 2880 ***g*** for 20 min to obtain white precipitate platelets. Phosphate‐buffered saline solution was used to wash the platelets, which were subsequently re‐suspended in RNAlater (Thermo Fisher Scientific, Waltham, MA, USA) and then frozen at −20 °C.

### RNA extraction

Total platelet RNA was extracted using the RNeasy Micro Kit (cat. no. 74004; Qiagen, Hilden, Germany) in accordance with the manufacturer’s instructions. Total RNA was qualified and quantified using model 2100 Bioanalyzer (Agilent, Santa Clara, CA, USA) and a Qubit RNA BR assay kit (Q10211; Invitrogen, Carlsbad, CA, USA), respectively. RNA with RIN ≥ 7 was considered high‐quality.

### PolyA‐mRNA purification and fragment

In accordance with the manufacturer’s instructions, poly‐(A)‐containing mRNA was purified using oligo (dT) magnetic beads (Thermo Fisher Scientific), as well as Oligotex mRNA Kits (Qiagen). Then, the purified products were fragmented at 94 °C for 15 min. The first‐strand cDNA was synthesized using the fragmented and primed mRNA, as well as ProtoScript reverse transcriptase (New England Biolabs, Ipswich, MA, USA) and the program was 25 °C for 10 min, 42 °C for 50 min and 70 °C for 15 min. The second‐strand cDNA was synthesized using the first‐strand cDNA and NEBNext second‐strand synthesis enzyme mix, and the program was 16 °C for 1 h. Then, the end repair/dA‐tail was performed by using NEBNext End Prep Enzyme Mix (New England Biolabs) and the program was 20 °C for 30 min, followed by 65 °C for 30 min. Adaptor ligation was also performed using dA‐Tailed cDNA, blunt/TA Ligase master mix and diluted NEBNext adaptor, and the program was 20 °C for 15 min, followed by 37 °C for 15 min. After end repair process and ligation of adapters, the cDNA fragments were amplified by using PCR Master Mix (Thermo Fisher Scientific). The PCR products were quantified using a Qubit DNA HS Assay Kit (Q32854; Invitrogen). The fragment size was detected using a model 2100 bioanalyzer chip (Agilent) and the concentration was analyzed using the KAPA kit (catalog. no. kk4602; Roche, Basel, Switzerland). The mixed library was stored at −80 °C.

### Study of generated data

The FASTQ‐files of raw data were aligned in RNA‐Seq, as reported previously [21]. Briefly, quality trimming of the 5′‐end and removal of sequence adapters were performed using Trimmomatic, version 0.22 [22] on the RNA‐Seq reads. Then, using STAR, version 2.5.1b [23], the reads were mapped to the Ensembl version 91 of the human reference genome. The summary of only reads spanning the introns was prepared using HTSeq, version 0.6.0 [24], applying the union intersection of uniquely aligned reads, and guided by version 75 of Ensembl gene annotation. Subsequently, all statistical and analytical evaluations were carried out in r, version 3.3.0, and r‐studio, version 0.99.902 (R Foundation for Statistical Computing, Vienna, Austria). Analysis of differential expression was carried out using the edgeR package, version 1.10.1 (https://bioconductor.org), for colorectal cancer and control groups. The read counts were corrected and were changed to counts‐per‐million, translated in the log_2_ format and multiplied by the TMM‐normalization factor determined by the calcNormFactors‐function of edgeR [25]. The statistically significant values selected for RNAs were those that were rectified for multiple hypotheses testing (false discovery rate; *P* < 0.05).

### GO and KEGG enrichment analysis

GO is a structured, controlled vocabulary for systematic analysis of gene function at the molecular and cellular levels [26]. KEGG is a collection of databases for biological interpretation of large‐scale datasets generated by transcriptome sequencing [27]. GO and KEGG pathway enrichment analysis was performed to functionally assign DEGs to specific terms and pathways via the r package clusterProfiler.

### Database mining

Raw data from the microarray dataset GSE30566 were downloaded from the Gene Expression Omnibus database (https://www.ncbi.nlm.nih.gov/geo) and normalized with GeneSpring software (Agilent) as log_2_ values. The normalized data were compared with our platelet RNA‐Seq data to select co‐DEGs using VENNY, version 2.1 (https://bioinfogp.cnb.csic.es/tools/venny). GO and KEGG enrichment analyses were performed again to functionally cluster the co‐GDTLs.

### Protein–protein interaction network analysis

To identify and understand functional protein interactions in human cells, as well as to systematically investigate the molecular mechanism of disease and identify new drug targets, we used STRING database (https://version11.string‐db.org) [28] to search known protein interactions and predict protein interactions and constructed a protein–protein interaction network.

### Statistical analysis

Student’s *t*‐test was performed using spss, version 21.0 (IBM Corp., Armonk, NY, USA). Receiver operating characteristic (ROC) curve drawing, the Pearson related test and other data visualizations were all carried out on the r studio platform. Data are presented as the mean ± SD of triplicate samples.

## Results

### Sample quality assessment

The platelet transcripts of 10 healthy individuals, three CKD patients and five DKD patients were quantitatively analyzed by the RNA‐Seq technique. On average, each of the 18 libraries produced 38.62 million reads. With FastQC (http://www.bioinformatics.babraham.ac.uk/projects/fastqc) evaluating the quality of sequencing reads, the results showed that the percentage of bases with Phred values were > 20 and 30 in the total bases, the values of *Q*_20_ (%) and *Q*_30_ (%) were more than 90%, the percentage of unidentifiable bases was less than or equal to 0.02%, and the sum of bases G and C accounts for about 50% of the total bases, indicating that the data output was well qualified. The result of reference sequence alignment showed that the total mapping percentage was more than 80%, whereas the data percentage of multiple mapping was < 10%, indicating that the data of this study was better than the reference genome and met the requirements of the next analyses.

### Transcriptome profiles in platelets of healthy controls are distinct from platelets of patients

**Fig. 2:**
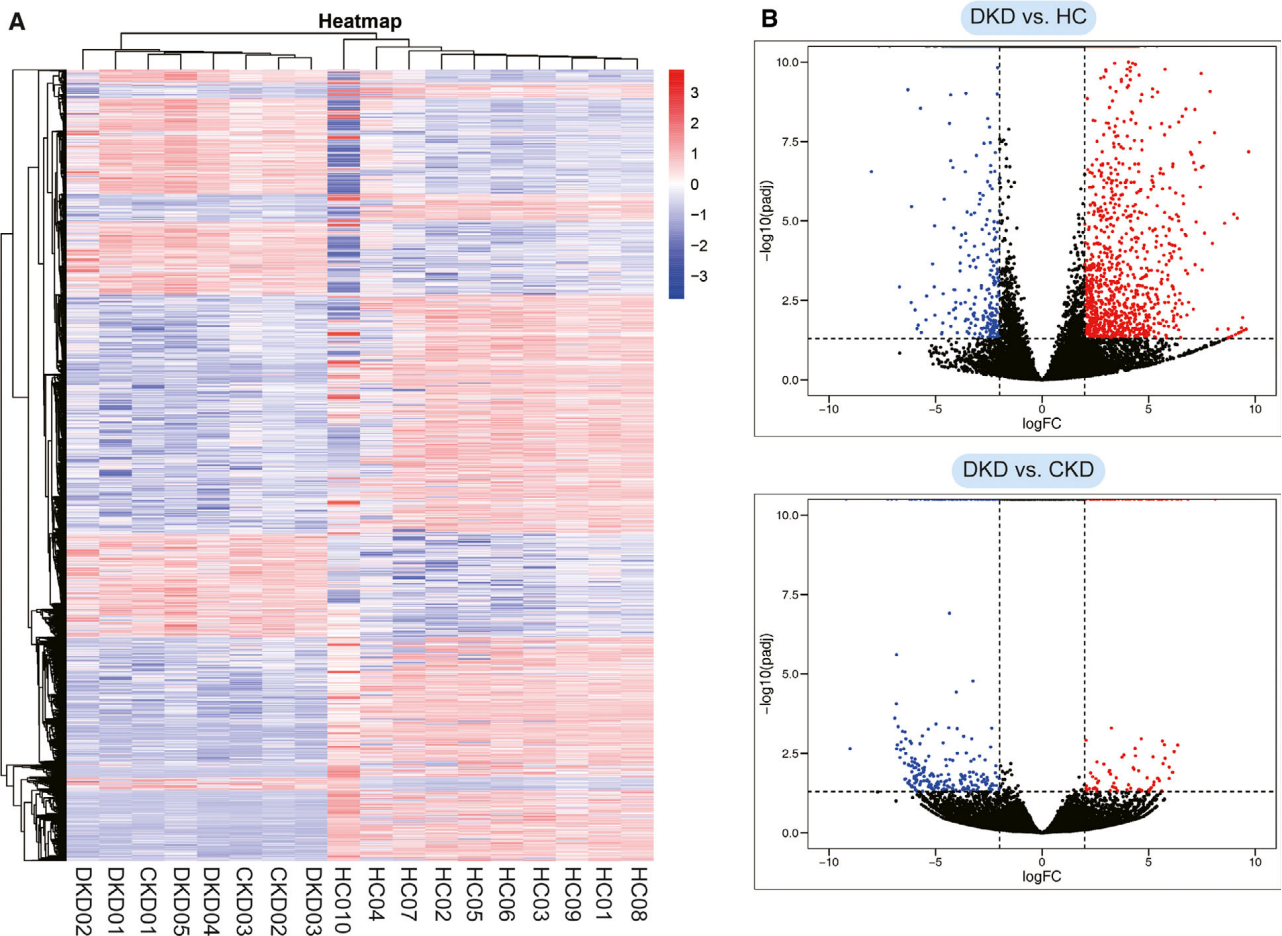
Gene expression profile of DKD, CKD and HCs. (A) Cluster analysis of DEGs and the heat map drawing according to DEG expression. Red for higher level, blue for a lower level. (B) Maps of the volcano drawn by all the DEGs. The dotted line represents the setting condition: |log_2_ fold change| ≥ 2 (vertical dotted line) and *P* < 0.05 (horizontal dotted line). Red represents significantly up‐regulated DEGs; blue represents significantly down‐regulated DEGs; black represents genes that do not meet the set conditions.

The results of 18 platelet sequencing samples were analyzed by systematic cluster analysis and heat mapping. As shown in Fig. 2A, the heat map results remarkably vary for gene transcriptions between the disease group and the HC group, although there were few differences between CKD and DKD. The volcano map was used to visualize GDTLs in HCs compared to DKD patients and CKD patients compared to DKD patients, with *P* < 0.05 and |log_2_ fold change| ≥ 2 (Fig. 2B). Specifically, 2097 GDTLs were identified in the DKD and HC groups, with 396 genes up‐regulated and 1701 genes down‐regulated. In the DKD and CKD groups, 462 GDTLs were identified, of which 317 genes were up‐regulated and 145 genes were down‐regulated. The top 10 up‐ and down‐regulated GDTLs in both groups are listed in descending order of |log_2_ fold change| values in Table 3.

**Table 3:** Top 10 up‐regulated and down‐regulated genes for DKD vs. HC and DKD vs. CKD.

Group	AccID	Gene name	log_2_FC	*P*‐value	Trend
DKD vs. HC	ENSG00000236894	AL160287.1	−22.93631683	7.62 × 10^−13^	Down
DKD vs. HC	ENSG00000228056	CFL1P3	−22.93072315	7.72 × 10^−13^	Down
DKD vs. HC	ENSG00000270093	AP000473.2	−22.92620774	7.80 × 10^−13^	Down
DKD vs. HC	ENSG00000213754	AL356317.1	−22.91959247	7.92 × 10^−13^	Down
DKD vs. HC	ENSG00000278988	AL356490.1	−22.9154346	7.99 × 10^−13^	Down
DKD vs. HC	ENSG00000101746	NOL4	−22.91329386	8.03 × 10^−13^	Down
DKD vs. HC	ENSG00000169154	GOT1L1	−22.91329386	8.03 × 10^−13^	Down
DKD vs. HC	ENSG00000199845	RNA5SP375	−22.91329386	8.03 × 10^−13^	Down
DKD vs. HC	ENSG00000201300	SNORD115‐27	−22.91329386	8.03 × 10^−13^	Down
DKD vs. HC	ENSG00000205667	ARSH	−22.91329386	8.03 × 10^−13^	Down
DKD vs. HC	ENSG00000271043	MTRNR2L2	8.196865419	8.75 × 10^−38^	Up
DKD vs. HC	ENSG00000164687	FABP5	8.008051887	4.66 × 10^−9^	Up
DKD vs. HC	ENSG00000210156	MT‐TK	6.68941899	5.92 × 10^−5^	Up
DKD vs. HC	ENSG00000170891	CYTL1	6.680930183	0.030197128	Up
DKD vs. HC	ENSG00000198868	MTND4LP30	6.296012728	7.69 × 10^−12^	Up
DKD vs. HC	ENSG00000269028	MTRNR2L12	6.25398937	4.11 × 10^−18^	Up
DKD vs. HC	ENSG00000229807	XIST	6.141886561	0.000232031	Up
DKD vs. HC	ENSG00000244921	MTCYBP18	6.123859794	8.00 × 10^−8^	Up
DKD vs. HC	ENSG00000160791	CCR5	5.958165204	0.000452019	Up
DKD vs. HC	ENSG00000122861	PLAU	5.866520384	0.002593308	Up
DKD vs. CKD	ENSG00000260804	LINC01963	−6.365999331	0.000902434	Down
DKD vs. CKD	ENSG00000168646	AXIN2	−6.198352713	0.001455783	Down
DKD vs. CKD	ENSG00000126861	OMG	−6.129516793	0.006457074	Down
DKD vs. CKD	ENSG00000143502	SUSD4	−6.12080688	0.006435488	Down
DKD vs. CKD	ENSG00000132744	ACY3	−5.966452952	0.004423797	Down
DKD vs. CKD	ENSG00000089723	OTUB2	−5.965811853	0.010085279	Down
DKD vs. CKD	ENSG00000280385	AP000648.3	−5.77485476	0.002340397	Down
DKD vs. CKD	ENSG00000154065	ANKRD29	−5.745925825	0.045825865	Down
DKD vs. CKD	ENSG00000162490	DRAXIN	−5.740880972	0.000874395	Down
DKD vs. CKD	ENSG00000161328	LRRC56	−5.69520794	0.04963958	Down
DKD vs. CKD	ENSG00000229807	XIST	9.009742697	0.001161334	Up
DKD vs. CKD	ENSG00000162598	C1orf87	7.714297968	0.027241706	Up
DKD vs. CKD	ENSG00000276566	IGKV1D‐13	6.909868648	0.00012734	Up
DKD vs. CKD	ENSG00000282608	ADORA3	6.837508535	4.50 × 10^−5^	Up
DKD vs. CKD	ENSG00000147647	DPYS	6.829925687	1.28 × 10^−6^	Up
DKD vs. CKD	ENSG00000108691	CCL2	6.828087851	0.001177822	Up
DKD vs. CKD	ENSG00000128567	PODXL	6.788531091	0.000893057	Up
DKD vs. CKD	ENSG00000134955	SLC37A2	6.754352046	0.000234756	Up
DKD vs. CKD	ENSG00000161888	SPC24	6.669035062	0.002202706	Up
DKD vs. CKD	ENSG00000129038	LOXL1	6.633684803	0.001268092	Up

### GO and KEGG pathway enrichment analysis

**Fig. 3:**
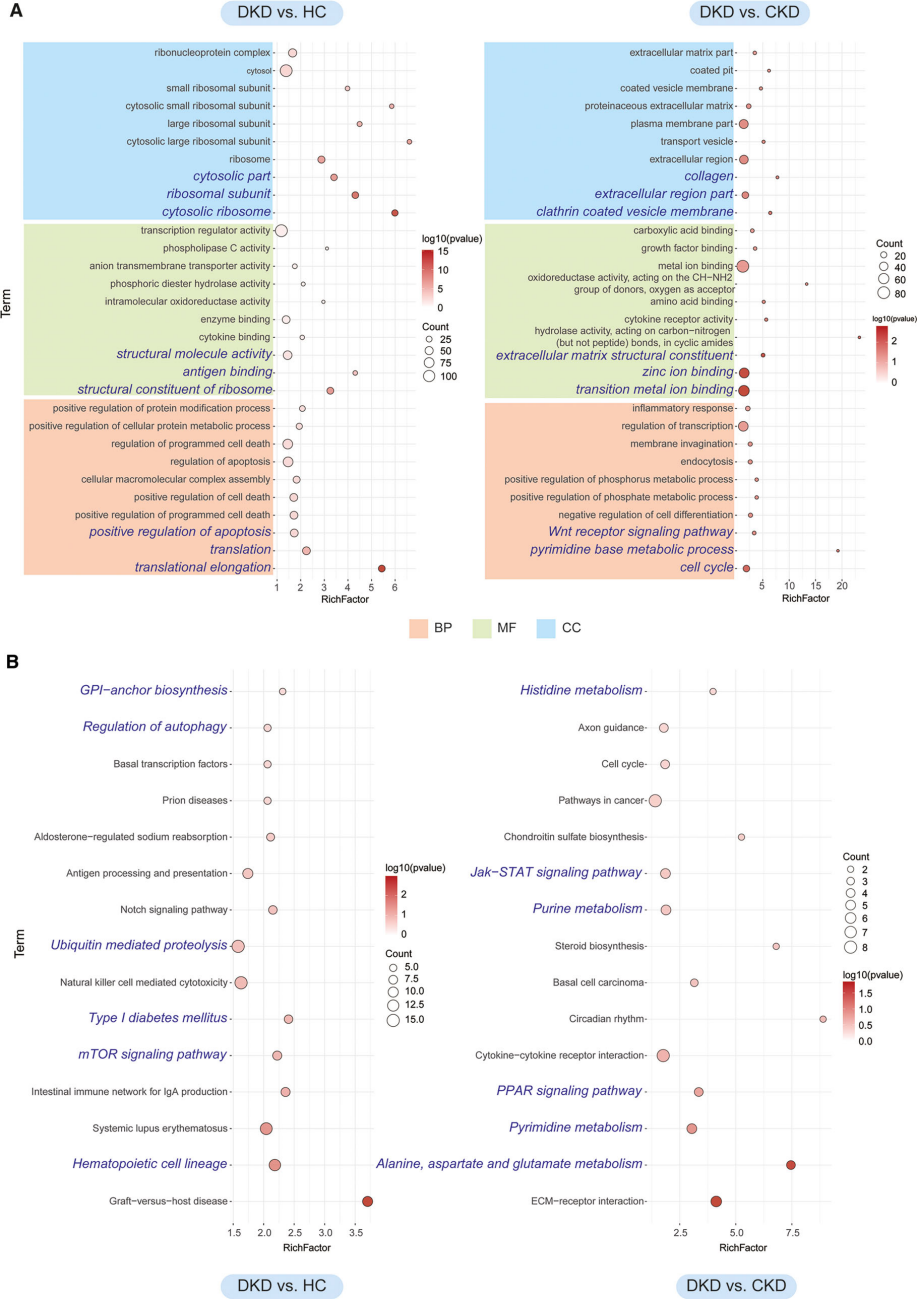
KEGG and GO enrichment pathway of DEGs in the DKD, CKD and HC groups. (A) Top 10 terms of MF, CC and BP (biological process) enriched by GO in ‘DKD vs. HC’ and ‘DKD vs. CKD’ (*P* < 0.05). The blue font represents the most significantly enriched term. The *y*‐axis on the left represents the GO term and the *x*‐axis indicates the ‘enrich factor’ represented by the ratio of DEGs numbers to the total annotated gene numbers of each term. The lower the *P*‐value, the more significant the enrichment. Low *P*‐values (*P* < 0.05) are shown in the red circle and high *P*‐values (*P* > 0.05) are shown in the white circle. (B) Top 15 enriched KEGG pathways in ‘DKD vs. HC’ and ‘DKD vs. CKD’. The blue font represents 11 valuable metabolic pathways. The *y*‐axis on the left represents KEGG pathways and the *x*‐axis indicates the ‘enrich factor’ represented by the ratio of DEGs numbers to the total annotated gene numbers of each pathway. The lower the *P*‐value, the more significant the enrichment. Low *P*‐values (*P* < 0.05) are shown in the red circle and high *P*‐values (*P* > 0.05) are shown in the white circle.

To understand more about the roles of the GDTLs between DKD with CKD patients, or DKD patients with HCs, GDTLs were assigned to GO terms, including biological processes (BP), cellular components (CC) and molecular function (MF) terms. As shown in Fig. 3A, In the DKD and HC groups, the most enriched BP terms were translational elongation, translation and positive regulation of apoptosis. The most enriched MF terms were cytosolic ribosome, ribosomal subunit and cytosolic part. The most enriched CC terms were cytosolic ribosome, ribosomal subunit and cytosolic part. In the DKD and CKD groups, the most enriched BP terms were cell cycle, pyrimidine base metabolic process and WNT receptor signaling pathway. The most enriched MF terms were transition metal ion binding, zinc ion binding and extracellular matrix structural constituent. The most enriched CC terms were clathrin‐coated vesicle membrane, extracellular region part and collagen.

To explore the pathways involved in the development of CKD and DKD, the GDTLs were submitted to KEGG analysis. In the DKD and HC groups, we selected six meaningful pathways, which were Hematopoietic cell lineage, Mammalian target of rapamycin (mTOR) signaling pathway, Type I DM, Ubiquitin mediated proteolysis, Regulation of autophagy and Glycosylphosphatidylinositol (GPI)‐anchor biosynthesis. In the DKD and HC groups, we selected five meaningful pathways, which were Alanine, Aspartate and glutamate metabolism, Pyrimidine metabolism, peroxisome proliferator‐activated receptor (PPAR) signaling pathway, Janus kinase‐signal transducer and activator of transcription (JAK‐STAT) signaling pathway and Histidine metabolism. The first 15 pathways in both groups are listed in Fig. 3B.

### Date mining from microarray datasets

**Fig. 4:**
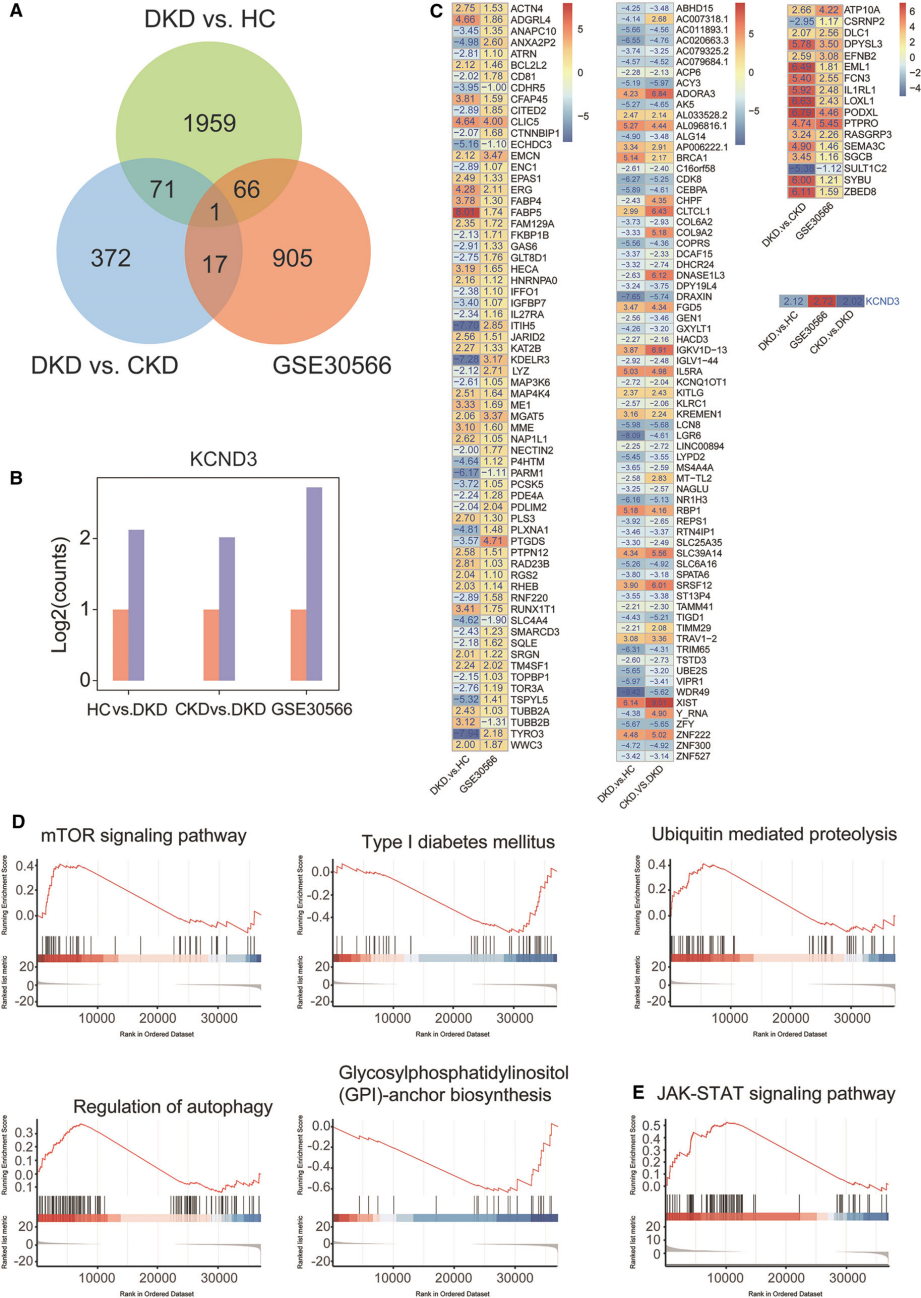
Overlapped DEGs in the GSE30566 database and platelets RNA‐Seq data. (A) Venn diagram of DEG distribution. Common and specific DEG numbers from different combinations displayed in the overlapping and non‐overlapping regions, respectively. (B) The bar chart of the expression level of co‐expression gene *KCND3* in the data set. The *x*‐axis represents the data set, the red bar represents the control group and the blue bar represents the observation group. The *y*‐axis represents the relative expression of genes. (C) Heat maps drawn using 66 DEGs (overlapping DKD vs. CKD and GSE30566), 17 DEGs (overlapping DKD vs. HC and GSE30566) and 71 DEGs (overlapping DKD vs. HC and DKD vs. CKD). (D) In DKD vs. HC, GSEA enrichment analysis of the mTOR signaling pathway, Type I DM, Ubiquitin mediated proteolysis, Regulation of autophagy and GPI‐anchor biosynthesis was carried out. (E) In DKD vs. CKD, GSEA enrichment analysis of the JAK‐STAT signaling pathway was carried out.

To improve our understanding and acquire further information that was relevant to CKD and DKD, we searched the Gene Expression Omnibus database for the datasets similar to renal diseases. Fortunately, we found the GSE30566 dataset, a microarray analysis dataset for identifying gene expression profiles in human CKD, which included 53 renal biopsy samples from CKD patients and eight normal renal biopsy samples from healthy individuals. Among this set, 909 down‐regulated and 80 up‐regulated signatures were identified. We carried out co‐expression analysis with this dataset compared to our data from the platelet RNA‐Seq. Finally, by drawing the Venn diagram for analysis, we visualized the co‐expression gene *KCND3* (*P* < 0.05, log_2_ fold change ≥ 1) (Fig. 4A). Some recent studies have shown that the mutation of *KCND3* is closely related to cerebellar ataxia, Brugada syndrome and long QT syndrome [29, 30].

A bar chart of *KCND3* changes in all datasets is shown in Fig. 4B. According to the Venn plot, heat maps were drawn using 66 GDTLs (overlapping CKD and DKD group and GSE30566), 17 GDTLs (overlapping HC and DKD group and GSE30566) and 71 GDTLs (overlapping the two groups), as shown in Fig. 4C. According to the GSEA enrichment analysis of all the genes enriched by HCs vs. DKD, we selected five pathways: mTOR signaling pathway, Type I DM, Ubiquitin mediated proteolysis, Regulation of autophagy, GPI‐anchor biosynthesis. As shown in Fig. 4D, mTOR signaling pathway, Ubiquitin mediated proteolysis and Regulation of autophagy are down‐regulated pathways. According to the GSEA enrichment analysis of all the genes enriched by CKD vs. DKD shown in Fig. 4E, the JAK‐STAT signaling pathway is down‐regulated.

### Diagnostic potential of crucial genes and correlation analysis between nine key biomarkers

**Fig. 5:**
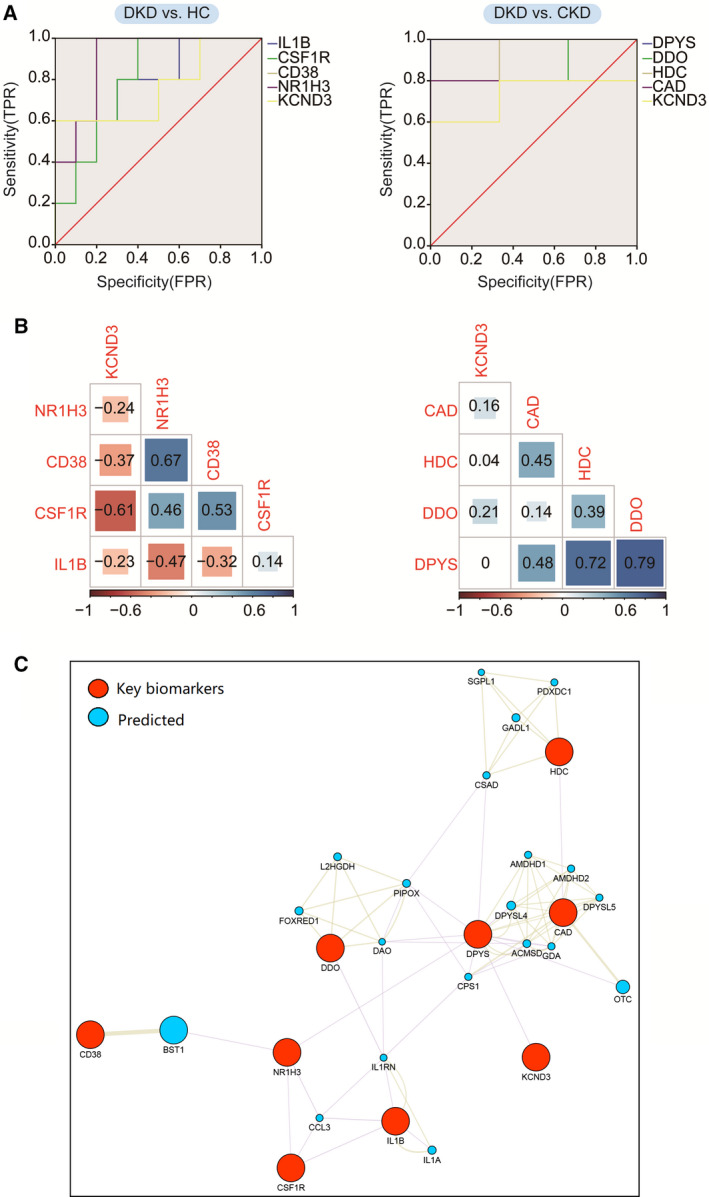
The biomarkers were analyzed by the PPI network, ROC and a Pearson correlation test. (A) The ROC curves of nine key biomarkers are plotted and the diagonal (red) represents AUC = 0.5. (B) Nine key biomarkers were tested for Pearson correlation. Red represents a negative correlation, blue represents a positive correlation and the number in the grid is the correlation coefficient. (C) The nine key biomarkers were analyzed by PPI network enrichment analysis. Red symbols represent the nine key biomarkers and blue symbols represent genes predicted to have interactions with the nine key biomarkers.

Next, ROC analysis (Fig. 5A and Table 4) was used to further verify the diagnostic potential of nine key biomarkers: *IL‐1B*, *CD‐38*, *CSF1R*, *PPARG*, *NR1H3*, *DDO*, *HDC*, *DPYS* and *CAD*. The results showed that, except for *KCND3*, the area under the curve (AUC) values of the other eight key biomarkers were all > 0.8, which means that the biomarkers we selected have good applicability.

**Table 4:** The AUC values of nine key biomarkers are calculated by drawing the ROC curve.

Group	Name	AUC
DKD vs. HC	IL1B	0.80
DKD vs. HC	CSF1R	0.80
DKD vs. HC	CD38	1.00
DKD vs. HC	NR1H3	0.90
DKD vs. CKD	DPYS	1.00
DKD vs. CKD	DDO	0.87
DKD vs. CKD	HDC	0.93
DKD vs. CKD	CAD	0.80
DKD vs. HC & DKD vs. CKD	KCND3	0.76 & 0.73

**Fig. 6:**
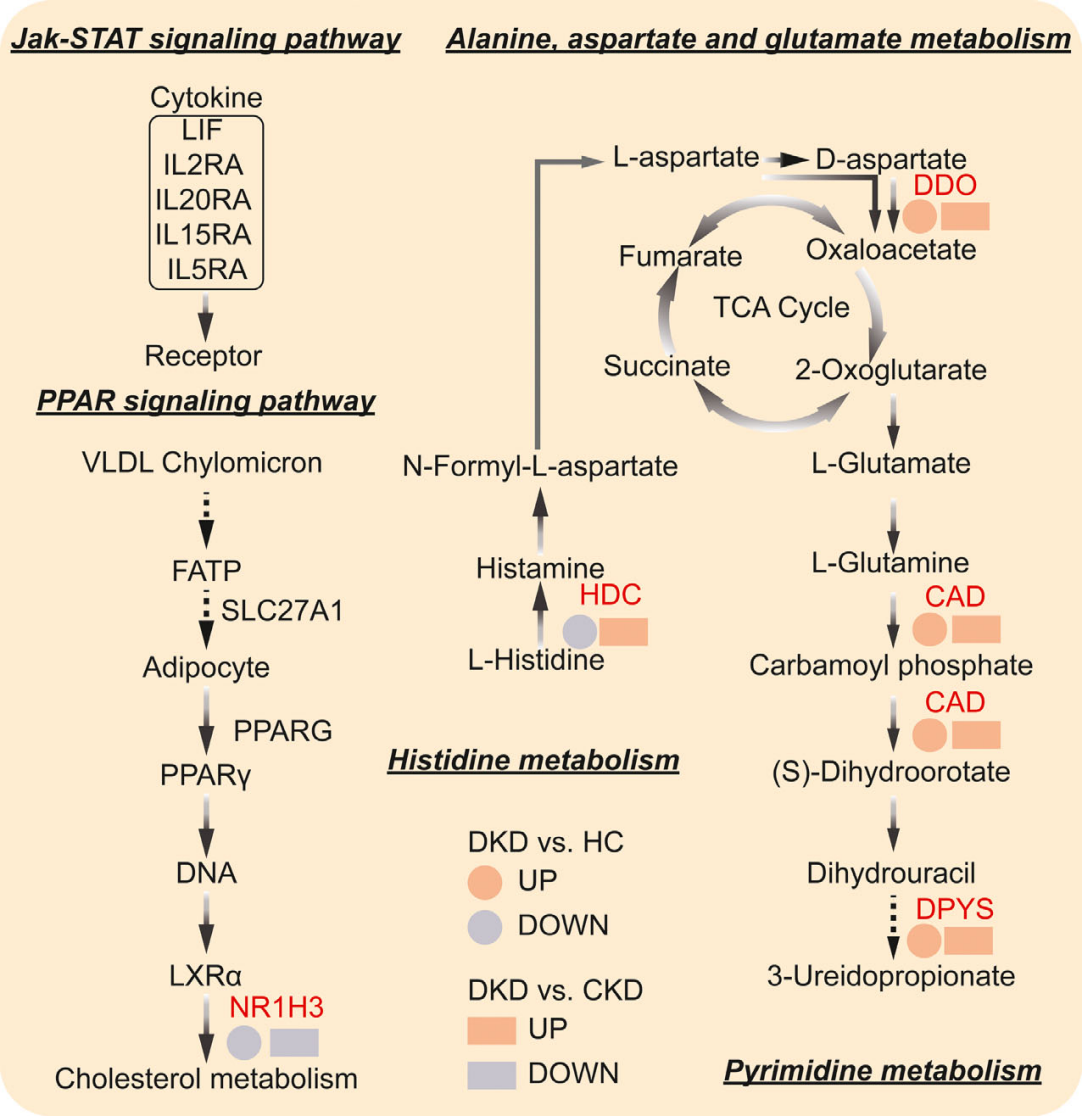
The simplified models of Alanine, aspartate and glutamate metabolism, Pyrimidine metabolism, PPAR signaling pathway, JAK‐STAT signaling pathway and Histidine metabolism.

In addition, we carried out Pearson rank correlation analysis (Fig. 5B) among the nine key biomarkers. The Pearson correlation coefficients of *r* > 0.6 and *r* ≤ −0.6 represent a significant positive correlation and negative correlation, respectively. We found that, in the HC and DKD groups, there was a positive correlation between *CD‐38* and *NR1H3* (*r* = 0.67) and a negative correlation between *CSF1R* and *KCND3* (*r* = −0.61). In addition, in the CKD and DKD groups, we found that *DPYS* was positively correlated with *HDC* (*r* = 0.72) and *ODD* (*r* = 0.79). Moreover, protein–protein analysis (PPI) analysis is an effective way of identifying new drug or therapeutic targets for various diseases [31, 32]. The PPI network analysis that we carried out showed the tight relationships of these biomarkers (Fig. 5C). We further visualized the relationships on pathway levels (Fig. 6).

## Discussion

Although eGFR and albuminuria have been the main biomarkers for DKD diagnosis in the clinic, DKD patients in the very early stage are always ignored. One reason is that changes of eGFR in these patients are not so remarkable [33]. Although DKD patients present proteinuria and impaired kidney function in the setting of diabetes with a distinct histopathological pattern of GBM thickening, mesangial matrix expansion, nodular glomerulosclerosis and arteriolar hyalinosis, kidney biopsy is not so frequently performed in patients with DM, which was considered to be dispensable for kidney disease patients [7, 8, 9, 34]. Studies aiming to identify potential biomarkers were performed in the past many years, with some progression being achieved. For example, the relationship between TNF–TNFR signaling and DKD progression was revealed, which is associated with inflammation [1, 35, 36, 37]. Inflammation has been reported to be involved in DKD [34, 35]. By coincidence, we identified *IL‐1B* and *CD‐38* to be down‐regulated in CKD patients and HCs compared to DKD patients, respectively, which makes them the potential biomarkers with respect to DKD progression. This result suggests that our RNA‐Seq analyses are reliable.

By comparing the data of platelet RNA‐Seq between DKD and HCs, we found some interesting pathways, such as Hematopoietic cell lineage, mTOR signaling pathway, Type I DM, Ubiquitin mediated proteolysis, Regulation of autophagy and GPI‐anchor biosynthesis. These pathways may provide a reference for the treatment of DKD. Diabetic glomerulosclerosis is still the cause of CKD in most patients with type 1 and type 2 diabetes [38]. Studies have shown that renal fibrosis can be alleviated by inhibiting the phosphoinositide 3‐kinase/Akt/mTOR signaling pathway [39]. Some studies have shown that the changes of gene expression levels in regulation of autophagy and Ubiquitin mediated proteolysis pathway are related to the consumption mechanism of skeletal muscle protein hydrolysis in CKD patients, such as decreased protein synthesis or increased degradation [40, 41, 42, 43]. Many eukaryotic proteins use GPI anchor to bind to the membrane. Although mammalian cells can survive without GPI anchors, a lack of hematopoietic cells can result in hemolytic disease, namely paroxysmal nocturnal hemoglobinuria [44]. Interestingly, GPI‐anchor biosynthesis was enriched as a pathway with respect to the treatment of CKD in metabonomics studies of rat CKD models [45]. The most valuable pathway of concern is Hematopoietic cell lineage, which is the most significantly enriched pathway in DKD vs. HC. Anemia and thrombosis are common complications of CKD. However, an erythropoiesis stimulant in anemic CKD patients may increase thrombotic activity by increasing hemoglobin levels or via other mechanisms (such as increased platelet reactivity and endothelial activation). Functional differences in the *IL‐1* family play an important role in CKD [46]. Our study found that the expression of *IL‐1R2, IL‐1R1* and *IL‐1B* in platelets in the DKD group was significantly higher than that in the HC group. *IL‐1B* is a major inflammatory cytokine produced by a variety of cells, including renal parenchyma cells and infiltrating cells, and is the mediator of acute and chronic inflammation [47]. In the treatment of CKD disease, the inhibition of *IL‐1B* expression is accompanied by the inhibition of kidney inflammation [47, 48].

In the platelet RNA‐Seq data of DKD vs. CKD, we found that, in the JAK‐STAT signaling pathway, the expression of *IL‐2RA, IL‐20RA, IL‐15RA* and *IL‐5RA* was significantly increased, whereas the expression of *ILF* was significantly decreased in DKD group compared to the CKD group (Fig. 6). The protein expression products of these genes are involved in cytokine–cytokine receptor interaction, and the differential expression of these genes in platelets may indicate that the level of inflammation in patients with DKD is different from that in CKD. The PPAR in the PPAR signaling pathway is a nuclear hormone receptor activated by fatty acids and their derivatives. There are three subtypes of PPAR (PPAR‐α, β/δ and γ), of which PPAR‐γ promotes adipocyte differentiation, thus increasing blood glucose uptake of [49]. The differential expression of gene *PPARG* is the main factor for the change of *PPAR‐γ* expression *in vivo*. Interestingly, we also found differential expression of *NR1H3* in cholesterol metabolism downstream. *NR1H3* is a key gene regulating liver X receptor α (LXR α). LXR α comprises a sterol‐regulated transcription factor that plays an important role in atherosclerosis by integrating cholesterol homeostasis and immunity [50]. Studies have shown that disrupting LXR α phosphorylation can regulate atherosclerosis by inducing macrophage proliferation [51]. In the present study, in comparison with the CKD group, we found that the expression of the *PPARG* gene in the platelet of DKD group was significantly increased, whereas the expression of *NR1H3* was significantly decreased, with a |log_2_ fold change| > 4.78 for both. This shows that is reasonable to propose *PPARG* and *NR1H3* as important targets for distinguishing between DKD and CKD.

As shown in Fig. 4B, we found that *KCND3* is the only up‐regulated gene in DKD patients compared to CKD, HCs or CKD‐related dataset GSE30566, which makes it a key target for the diagnosis and treatment of DKD patients. Although the AUC value of *KCND3* is lower than 0.8, Pearson rank correlation analysis showed the significance of *KCND3* to be an important biomarker (with *r* = −0.61). In the ventricle, the *KCND3* gene encodes voltage‐gated rapid inactivation of the main pore‐forming α subunit of the type A potassium channel, and participates in a rapid cardiac transient outward potassium current (Ito), which plays a major role in the early repolarization phase 1 of cardiac action potential [52]. Most of the studies of *KCND3* focus on cerebellar ataxia, Brugada syndrome and long QT syndrome [53]. However, there are few studies concerning the importance of *KCND3* in the kidney. Our study shows that *KCND3* is the key biomarker for diagnosis of DKD patients, which needs to be confirmed in further research.

The present study depends on the RNA‐Seq of venous blood platelets in DKD or CKD patients. Despite the rapid development of sequencing techniques, the blood samples of DKD patients have been in the spotlight for many years. The search for new biomarkers of DKD has centered primarily on identifying analyses in urine and blood that improve the prediction of later established end points, and then the differentially expressed microRNAs and epigenetic modifications in kidney tissues [31, 54]. We noted that DKD progression in DM patients is genetically related and we first performed the RNA‐Seq for venous blood platelets of CKD or DKD patients. We identified several potential biomarkers for the diagnosis and treatment of DKD patients. We hope that our study will make it possible to detect and monitor DKD patients at an early stage, as well as prevent DKD progression effectively.

In summary, we have comprehensively identified 2097 GDTLs in the venous blood platelets of HCs vs. DKD and 462 GDTLs in platelets of CKD vs. DKD for the first time, and identified nine potential biomarkers for DKD patients: *IL‐1B*, *CD‐38*, *CSF1R*, *PPARG*, *NR1H3*, *DDO*, *HDC*, *DPYS* and *CAD*. In addition, we have also annotated and analyzed the differential transcript profiles in RNA‐Seq and microarray datasets and found biomarker *KCND3* to be the only up‐regulated gene in DKD patients. In‐depth studies are necessary to reveal the function and mechanism of these potential genes in DKD diagnosis and therapies.

## Data accessibility

The datasets generated during and/or analyzed during the present study are available from the corresponding author upon reasonable request.

## Author contributions

XLZ and HMJ conceived and designed the research. All authors conducted the experiments and analyzed data. BLZ and XHY wrote the manuscript and all authors commented on previous versions of the manuscript. All authors have read and approved the final version of the manuscript submitted for publication.

## Conflict of interests

The authors declare that they have no conflicts of interest.
